# Diphtheria in London

**Published:** 1888-10-27

**Authors:** 


### The Hospital

A Weekly Institutional Journal of

**Science, Medicine, Nursing, and Philanthropy.**

*For Hospitals, Asylums, Medical Practitioners, Students, Nurses, Families, Parishes, Congregations, and the Charitable Public.*

Vol. V. No. 109 ] [October 27, 1888.

### DIPHTHERIA IN LONDON.

We have to congratulate the public and the general hospitals on the steps which the Local Government Board has at last taken with reference to the isolation of diphtheria in London. At a meeting of the Managers of the Metropolitan Asylums, held last Saturday, and in response to their repeated applications for permission to utilise the hospitals for infectious disease for the treatment of diphtheria, a communication was received from the Secretary of the Local Government Board conceding the point at issue, and giving full power to the Managers to proceed at once with their laudable intention. It is a singular fact that, although the rich Asylums Board was called into existence twenty-one years ago for the purpose, among other things, of isolating infectious fever and small-pox, it had no authority to deal with such a fatally infectious fever as diphtheria is admitted to be. The want of this power has been repeatedly brought under the notice of the two bodies mainly interested, more especially during recent years, when the ravages of the distemper became more marked, but both appeared equally helpless to solve the difficulty.

Less than two years ago the Asylums Board Managers appealed to the Local Government Board for fresh instructions, and received an answer that if diphtheritic cases were to be admitted into the infectious hospitals it would be necessary to have special legislation for the purpose, as the original Act of Parliament only referred to infectious fever and small-pox. According to Sir Wm. Jenner's interpretation of the term fever, it only comprises enteric, typhus, and scarlet fever, leaving diphtheria and measles out of the definition entirely. The subject has been again pressed on the notice of the authorities during the present year, supported by memorials from some of the leading hospitals, in such a way as to render it impossible to be further delayed. The Local Government Board has decided on applying to Parliament for a new Act empowering the Asylums Managers to deal with diphtheria in the same manner as they have successfully dealt with infectious fever and small-pox, and which they would have to deal with cholera should it ever re-visit us. Moreover, until this power is obtained, the Asylums Managers are at liberty to admit diphtheritic patients into the hospitals under their control, and the expenditure incurred in consequence will be sanctioned by the Board. Here we have a satisfactory solution of a question which has given much anxiety to the public, and especially to health officers and hospital authorities for many years.

Diphtheria is one of the numerous diseases attributable to filth, and consequently remediable, but, on account of its virulently contagious character, it seldom leaves a household without affecting more than one of its members, often the nurse, especially if she is young and emotional; and occasionally the doctor. On these grounds, combined with the fatal character of the malady, and often the immediate necessity for surgical interference, there are few distempers in which efficient isolation becomes more imperative, or is likely to be attended with more beneficial results. The general hospitals have hitherto borne the brunt of grappling with bad cases of the malady, and have assumed the responsibility of admitting or rejecting such when brought to them. The sufferers are for the most part children conveyed in street cabs or in their mothers' arms, and as a rule they are placed in the same wards as others not similarly affected. The dangers arising from such an admixture, although grave, are not so serious as might be apprehended, the spread of infection for the most part being confined to the immediate attendants. Now, London hospitals have had cause to mourn the loss of many young lives among their nurses and resident medical staff from contact with the disease; and, notwithstanding the coming departure from long established usage, it may be reasonably anticipated that it will be well-nigh impossible to banish diphtheria entirely from them. The reason of this is obvious enough. It has been long the custom of parents to retain their children affected by the disease in their own custody until there is imminent danger of death from suffocation, when, in a kind of frantic terror, they hurry them off to the nearest hospital, where it may be considered advisable to have the operation of tracheotomy performed as a forlorn hope to save life. It is not to be expected that under these distressing circumstances the hospital authorities would delay matters by despatching the sufferers by ambulance to the nearest infectious hospital, as they would inevitably do with small-pox and scarlet fever. On this account, if on no other, there is little reason to fear that the student would be deprived of the privilege of seeing and studying diphtheria if he has a mind to do so, and we are also informed that the Asylums Managers are disposed to give them every facility for clinical research at the hospitals under their control.

But in entering on the new department of their

ARMY MEDICAL LIBRARY

work, we are not sure that the gentlemen who comprise the Metropolitan Asylums Board are fully alive to the difficulties which they may have to encounter. We know less about the causation of diphtheria than we do of more contagious diseases, and there is much difference of opinion among medical men with regard to its true characteristics. Other affections of the throat and mouth, especially croup, and, probably, also the sore throat of scarlet fever, are frequently confounded with diphtheria; while the true complaint may be passed over in the absence of any well-marked symptoms. It will require a wise discrimination on the part of the resident medical officers of the Board to separate the infection from the less virulent complaint, which can only be gained by experience and a knowledge of the special characters of the prevailing epidemic. Special consideration must also be given to the age and character of the attendants, for it is a well-known experience that young women and young doctors are far more liable to suffer from the diphtheritic virus than those of mature years. We have no desire to see the installation of a new order of Sarah Gamps into the infectious hospitals, for there are plenty of respectable nurses well advanced in life who would readily undertake such duties, provided they were remunerated on a scale commensurate with the manipulative skill required, and the dangers which they must inevitably have to encounter. Whatever the difficulties, the Asylum Managers are ready to face them, and they have every reason to be gratified at their moral victory in obtaining the sanction of the Government to a scheme which they have long had at heart, and which may possibly be the means of stamping out in London not the least virulent of our modern plagues.

